# Soil Application of Zinc Fertilizer Increases Maize Yield by Enhancing the Kernel Number and Kernel Weight of Inferior Grains

**DOI:** 10.3389/fpls.2020.00188

**Published:** 2020-02-28

**Authors:** Dun-Yi Liu, Wei Zhang, Yu-Min Liu, Xin-Ping Chen, Chun-Qin Zou

**Affiliations:** ^1^ Key Laboratory of Efficient Utilization of Soil and Fertilizer Resources, College of Resources and Environment, Southwest University, Chongqing, China; ^2^ Key Laboratory of Plant-Soil Interactions, Ministry of Education, Center for Resources, Environment and Food Security, China Agricultural University, Beijing, China

**Keywords:** critical zinc concentration, pollen viability, kernel abortion, apical kernels, maize

## Abstract

Improving the development of inferior grains is important for increasing maize yield under high-density conditions. However, the effect of micronutrients, especially zinc (Zn), on the development of inferior grains and maize yield under field conditions has not been evaluated to date. A field experiment with six Zn application rates (0, 2.3, 5.7, 11.4, 22.7, and 34.1 kg/ha) was conducted to investigate the effects of soil application of Zn fertilizer on the development of inferior grains. Pollen viability was measured at the tasseling stage. The maize spike was divided into apical (inferior grain), middle, and basal sections for further measurement at harvest. Results showed that soil application of Zn fertilizer increased maize yield by 4.2–16.7% due to increased kernel number and weight in the apical, but not in the middle and basal sections. Zn application also significantly increased pollen viability at the tasseling stage. The critical Zn concentrations in shoots at the tasseling stage for obtaining high pollen viability and high kernel numbers of inferior grains were 31.2 and 35.6 mg/kg, respectively. Zn application also increased the 1,000-kernel weight of inferior grain due to high biomass accumulation. Furthermore, the grain Zn concentration of inferior grain with Zn application increased by 24.3–74.9% compared with no Zn application. Thus, soil application of Zn fertilizer successfully increased grain yield of maize by improving pollen viability, kernel number, and kernel weight of inferior grains (apical section), also contributing to grain Zn biofortification.

## Introduction

As a common staple food, fuel, and feed, maize (*Zea mays* L.) yields have continued to increase (Duvick, 2005; Nuss and Tanumihardjo, 2010). However, the yield gap of maize (the gap between potential and real yield) is still large. None of the major maize regions worldwide exceeds 70% of yield potential (Lobell et al., 2009).

A sufficient kernel number per spike and high kernel weight are important for guaranteeing a high maize yield at certain densities (Borrás and Otegui, 2001). One physiological basis for the improvement of modern maize hybrids is an increase in kernel number per unit area, which accounts for most of the variation in maize yield (Duvick, 1997). Therefore, increasing plant density in the newest hybrids is the main strategy for improving yield in maize (Ma et al., 2014; Di Matteo et al., 2016). For instance, the density of maize in the US Corn Belt has increased by about 1000 plants/ha/year over the last 50 years (Ma et al., 2014). However, excessively high density can lead to a decline in maize yield (Boomsma et al., 2009), mainly because the kernel numbers per spike often decline with increasing plant density (Sangoi, 2001), especially in the apical section (Chen et al., 2013).

Although zinc (Zn) is an essential micronutrient for plant growth, Zn input has received much less attention than nitrogen (N), phosphorus (P), or irrigation during the Green Revolution (Tilman et al., 2002; Mueller et al., 2012). However, nearly half of the cereal-growing areas worldwide have soils with low plant-available Zn. Therefore the application of Zn fertilizers is necessary in such soils to ensure cereal yield and grain Zn concentration (Cakmak, 2008). Many studies have demonstrated that the maize grain yield increases significantly with the application of Zn fertilizer to Zn-deficient soils (Abunyewa and Mercie-Quarshie, 2004; Potarzycki, 2010; Liu et al., 2017a). A better understanding of the physiological role of Zn fertilizer application in increasing the yield of maize is needed.

Due to sterility and their limited retranslocation of resources, maize grains located on the apical part of the ear often develop poorly, and are classified as inferior grains, while the middle and basal parts of the ear represent superior grains (Zhao et al., 2018). The differences have proven to be exacerbated under inappropriate cultivation conditions and environmental factors, often resulting in abortion of inferior grains and the formation of bald ear tips that significantly limit grain yield. Several studies have shown the response of inferior grains to various abiotic factors, such as high temperature (Edreira et al., 2011), shade (Setter et al., 2001), intra-specific competition (Pagano and Maddonni, 2007), and insufficient irrigation and nitrogen N (Pandey et al., 2000). Some reports also showed that a lack of Zn decreases pollen viability and leads to pollen sterility in maize (Sharma et al., 1990) and then to low kernel numbers. However, the critical Zn concentration in shoots to maintain high pollen viability and kernel number (especially in inferior grains) is not well known.

Maize inferior grains often have fewer kernels and lower biomass; thus, improving the development of these inferior grains could increase the maize grain yield. A way to improve inferior grains can thus be Zn fertilization, because a positive relationship between kernel number and the maize stem Zn content has been reported, whereas the absence of Zn induced barren ear tips (Mozafar, 1987; Potarzycki, 2010). Insufficient assimilation caused by abiotic stress, such as Zn deficiency, led to a shorter duration of linear grain-filling in later-growing kernels (Serrago et al., 2013). Improved maize shoot biomass with increasing Zn supply indicated that adequate Zn led to assimilation of the available supply (Liu et al., 2017a). Zinc management is vital to increasing pollen viability and Zn assimilation in maize and to guaranteeing grain development, especially in the apical section.

Therefore, this study aimed to quantify the effects of Zn fertilizer on pollen viability, kernel number, and grain weight in different sections of the maize spike, with focus on inferior grains, and explored how Zn application improved the development of inferior grains and contributed to yield increase in maize.

## Materials and Methods

### Field Location

Field experiments have been conducted at the Quzhou Experimental Station (36.9°N, 115.0°E) on the North China Plain since 2009. This study examined all data on yield components, pollen viability, biomass, and nutrients of maize in the 2012 cropping season. The total precipitation is 384 mm, the mean temperature is 25.2°C and the total sunshine duration is 640 h during this cropping season (from middle June to early October). The soil is a typical calcareous alluvial soil with the following characteristics: pH, 8.0 (1:2.5 w/v in water); organic matter concentration, 1.0%; total nitrogen concentration, 0.62 g/kg; Olsen phosphorus, 6.9 mg/kg; available potassium, 96 mg/kg; cation exchange capacity (CEC), 11.6 cmol/kg; CaCO_3_ concentration, 4.5%. The initial soil diethylenetriamine pentaacetate (DTPA)-extractable Zn concentration was 0.45 mg/kg, which indicated Zn deficiency (low, 0-0.5 mg/kg; medium, 0.51-0.8 mg/kg; high, > 0.8 mg/kg). The soil texture is silt loam soil with 7.9% of clay (<2 μm), 55.3% of silt (2–20 μm), and 36.8% of sand (20–2,000 μm).

### Experimental Design

The experiment involved a winter wheat–summer maize rotation system. The treatments included six application rates of Zn fertilizer before summer maize planting. The rates were 0, 2.3, 5.7, 11.4, 22.7, and 34.1 kg/ha of Zn (as 0, 10, 25, 50, 100, and 150 kg/ha of ZnSO_4_·7H_2_O, respectively). All plots were arranged in random blocks with four replications, and each plot was 75 m^2^ (15 m long x 5 m wide). A Zn fertilizer solution was sprayed on the soil surface just before sowing and then incorporated into the soil by disk plowing. Other nutrient supplements were standardized across treatments: 225 kg N ha^−1^, 75 kg P_2_O_5_ ha^−1^, 75 kg K_2_O ha^−1^ and no manure was applied. A compound fertilizer (N–P_2_O_5_–K_2_O: 15–15–15; 75 kg/ha) was disked into the soil before the maize was sown, and 150 kg N/ha (as urea) was applied at the six-leaf (V6) stage. The rate of N, P_2_O_5_, and K_2_O fertilization was determined according to the Guidelines for fertilization of major crops in China (Zhang et al., 2009), which indicated that when the target yield of maize is about 10–12 t/ha, the appropriate N, P_2_O_5_, and K_2_O application rate is 210–240, 75–90, and 75–90 kg/ha, respectively.

Maize (*Z. mays* L. cv. Zhengdan958) was planted in June 18 and harvested on October 5, 2012. The density was about 75,000 plants/ha, with a row spacing of 60 cm and plant spacing of 22.3 cm, which is a typical high-density regime in the North China Plain (Yan et al., 2017). Irrigation was applied before seedling emergence. Herbicides and pesticides were applied to control weeds and pests at the pre-emergence and 12-leaf (V12) stages, respectively. No obvious water, weed, or pest problems were observed during the experiment. Standard growth stages of maize were taken from Ritchie and Hanway (1982).

### Sampling and Analysis

Maize pollen grains were sampled at the beginning of the tasseling (VT) stage. Pollen viability was tested by staining with a 1% solution of 2,3,5-triphenyl tetrazolium chloride (Cook and Stanley, 1960). Shoot samples of four maize plants were collected at the 6-leaf (V6), 12-leaf (V12), tasseling (VT), milk (R3), and harvest (R6) stages. At harvest, all maize ears in a 12-m^2^ area (4 rows of approximately 2.4 × 5 m each) were harvested to determine the grain yield. Twenty spikes from 20 contiguous plants in one row were cut into three equal sections by length using a stainless steel knife (apical, middle, and basal) (Mozafar, 1990). The kernel number (KN) and kernel weight were determined for each section separately. Thousand kernel weight (TKW) were determined using the weight of three sets of 200 kernels in each section of the maize spikes.

All plants samples were rapidly washed with deionized water and then dried at 65°C in a forced-draft oven to a constant weight. The plant samples were ground with a stainless steel grinder and digested with HNO_3_–H_2_O_2_ in a microwave-accelerated reaction system (CEM; Matthews, NC, USA). The Zn concentrations in the digested solutions were determined by inductively coupled plasma–optical emission spectroscopy (OPTIMA 7300 DV; Perkin–Elmer, USA). IPE684 and IPE126 (Wageningen University, the Netherlands) were used as reference materials for grain and straw analysis, respectively.

### Calculations and Statistical Analysis

The change in yield of different spike sections was calculated as Y_treatment_–Y_Zn0_, where Y_treatment_ is the yield of different sections in each plot and Y_Zn0_ is the mean yield with no Zn application. The change in kernel numbers in the three sections was calculated similarly.

Before analysis, data normality and variance homogeneity were checked by SPSS 20.0 (*P* > 0.05). The effects of Zn application rate on grain yield, pollen viability, shoot biomass and Zn concentration in shoot and grain were evaluated by one-way analysis of variance (ANOVA). Means were separated by Fisher’s protected least significant difference (LSD) test at *P* < 0.05. Two-way repeated measures ANOVA was used to assess the effect of Zn application rate, spike section, and their interactions on the dependent variables. The relationships between the Zn concentrations in the plant and soil at the VT stage and between pollen viability and the number of kernels per spike were assessed by nonlinear regression using the NLIN procedure in SAS software (SAS 8.0, USA); the linear-with-plateau model (Cerrato and Blackmer, 1990) produced the best fit. SPSS 20.0 was used to perform path coefficient analysis to evaluate the contribution of kernel numbers in the apical section of maize spikes to grain yield and the relationships between yield and yield components in the three spike sections.

## Results

### Grain Yield, Kernel Number, and Kernel Weight in Three Sections of Maize Spikes

Zinc application significantly increased maize grain yield by 4.2–16.7% compared with no Zn treatment (**Table 1**). Both KN and TKW in the apical sections of maize spikes increased significantly with the amount of Zn applied, while the KN and TKW of the middle and basal sections were not significantly affected (**Table 1**). Compared with no Zn addition, the KN and TKW of the apical section of maize spikes increased by 19.3–54.5 and 2.14–7.30%, respectively. Both KN and TKW of the apical section of maize spikes were lower than those of the middle and basal sections (**Table 1**).

**Table 1:** Effects of Zn various application rates on grain yield, kernel number per spike, and 1,000-kernel weight of the apical, middle, and basal sections of maize spikes under field conditions.

Zn rate (kg/ha)	Grain yield (g/plant)	Kernel number			1,000-Kernel weight (g)		
		Apical	Middle	Basal	Apical	Middle	Basal
0	120 b	88.5 d	177 a	178 a	233 b	278 a	281 a
2.3	125 ab	105 cd	179 a	173 a	238 ab	284 a	286 a
5.7	128 ab	114 bc	176 a	181 a	240 ab	282 a	282 a
11.4	136 a	129 abc	182 a	180 a	246 ab	285 a	291 a
22.7	137 a	140 a	179 a	184 a	244 ab	282 a	285 a
34.1	140 a	136 ab	185 a	182 a	250 a	286 a	291 a
Source of variation							
Sections	–	***			***		
Zn	**	**			**		
Zn* section	–	ns			ns		

Values are the means of four replicates. Means in a column followed by the same letters are not significantly different at P < 0.05 according to Fisher’s LSD test. *** P< 0.001, **P < 0.01. ns, not significant.

Pearson’s correlation analysis showed that maize grain yield was positively correlated with the KN and TKW of the apical section of maize spikes. Further analysis indicated that the KN of the apical section explained 17% of the grain yield increase (**Table 2**).

**Table 2:** Relationships between yield and yield components in three sections of maize spikes based on Pearson’s correlation analysis.

	Grain yield	KN-A	KN-M	KN-B	TKW-A	TKW-M	TKW-B
Yield	1.000						
KN-A	0.453**	1.000					
KN-M	0.297 ^ns^	0.738***	1.000				
KN-B	0.272 ^ns^	0.771***	0.964***	1.000			
TKW-A	0.324*	0.566**	0.505**	0.505**	1.000		
TKW-M	0.155^ns^	0.547**	0.525**	0.565**	0.688***	1.000	
TKW-B	0.197^ns^	0.578**	0.610**	0.541**	0.751***	0.877***	1.000

Values in bold are the direct effects of KN-A on yield based on path coefficients; adjusted R^2^ = 0.17.

***P < 0.001, **P < 0.01, and *P < 0.05. ns, not significant.

KN-A, KN-M, and KN-B are the kernel numbers in the apical, middle, and basal sections, respectively; TKW-A, TKW-M, TKW-B are the 1,000-kernel weight in the apical, middle, and basal sections, respectively.

### Pollen Viability and Shoot Zn Concentration at the Tasseling Stage

**Figure 1:**
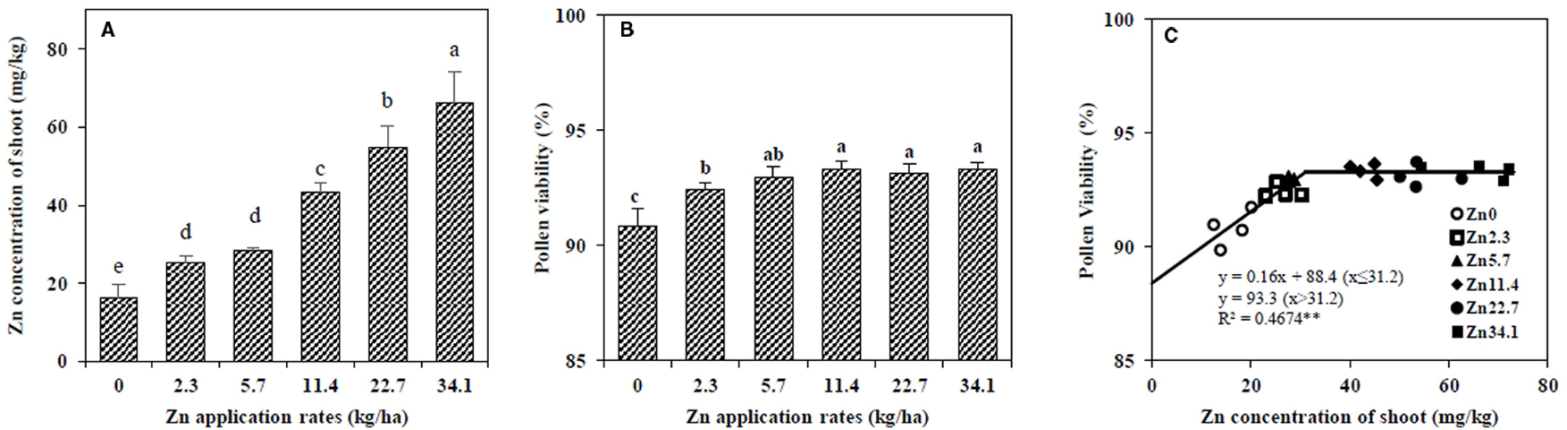
Effect of Zn application rate on the Zn concentration in the shoot **(A)** and pollen viability **(B)** in maize at the tasseling (VT) stage, and the relationship between pollen viability and Zn concentration in the shoot at the VT stage **(C)**. The pollen viability was scored according to staining level (pollen with bold red colour as viable and colourless as nonviable). The percentage of pollen viability was determined as the ratio of the number of viable (energetic) pollens to the total pollens number. Values are the average ± SE of four replicates. Means with the same letters are not significantly different at *P* < 0.05 according to Fisher’s least significant difference (LSD) test. ** *P* < 0.01.

At the VT stage, the shoot Zn concentration increased with higher amounts of Zn fertilizer (**Figure 1A**). Pollen viability increased with Zn application from 0 to 5.7 kg/ha, and then remained steady at higher Zn rates (**Figure 1B**). The linear-plateau model described the relationship between pollen viability and Zn concentration in maize shoots at the VT stage well. The critical shoot Zn concentration at which the pollen viability was the highest was 31.2 mg/kg (**Figure 1C**).

### Kernel Number in Three Maize Spike Sections and Grain Yield Were Correlated With Pollen Viability and Shoot Zn Concentration

**Figure 2:**
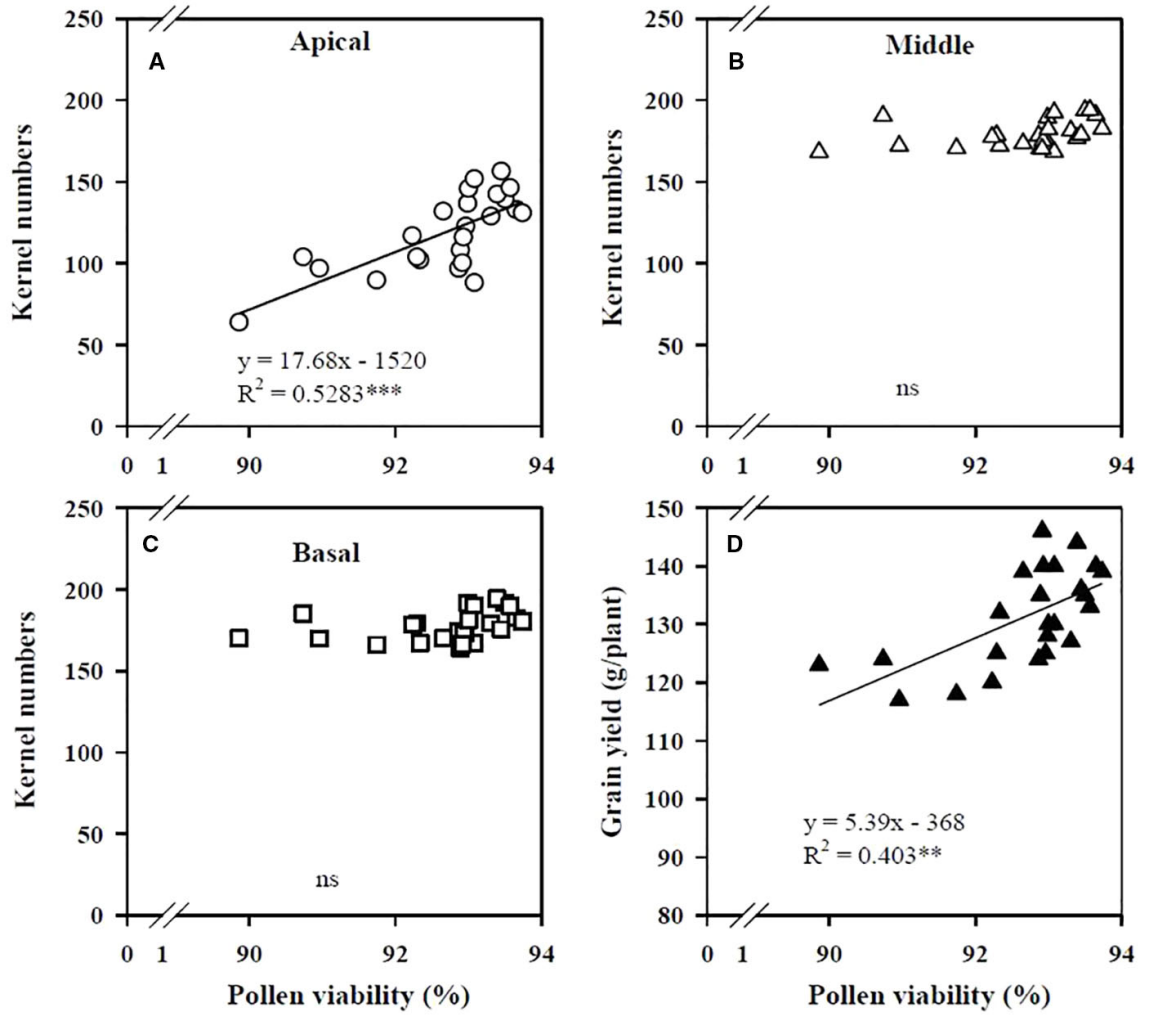
Correlations of the kernel numbers of the apical **(A)**, middle **(B)**, and basal **(C)** sections of maize spikes and maize grain yield **(D)** with pollen viability at the tasseling (VT) stage in the field. The percentage of pollen viability was determined as the ratio of the number of energetic pollens to the total pollens number. ***P < 0.001 and ** *P* < 0.01. ns, not significant.

**Figure 3:**
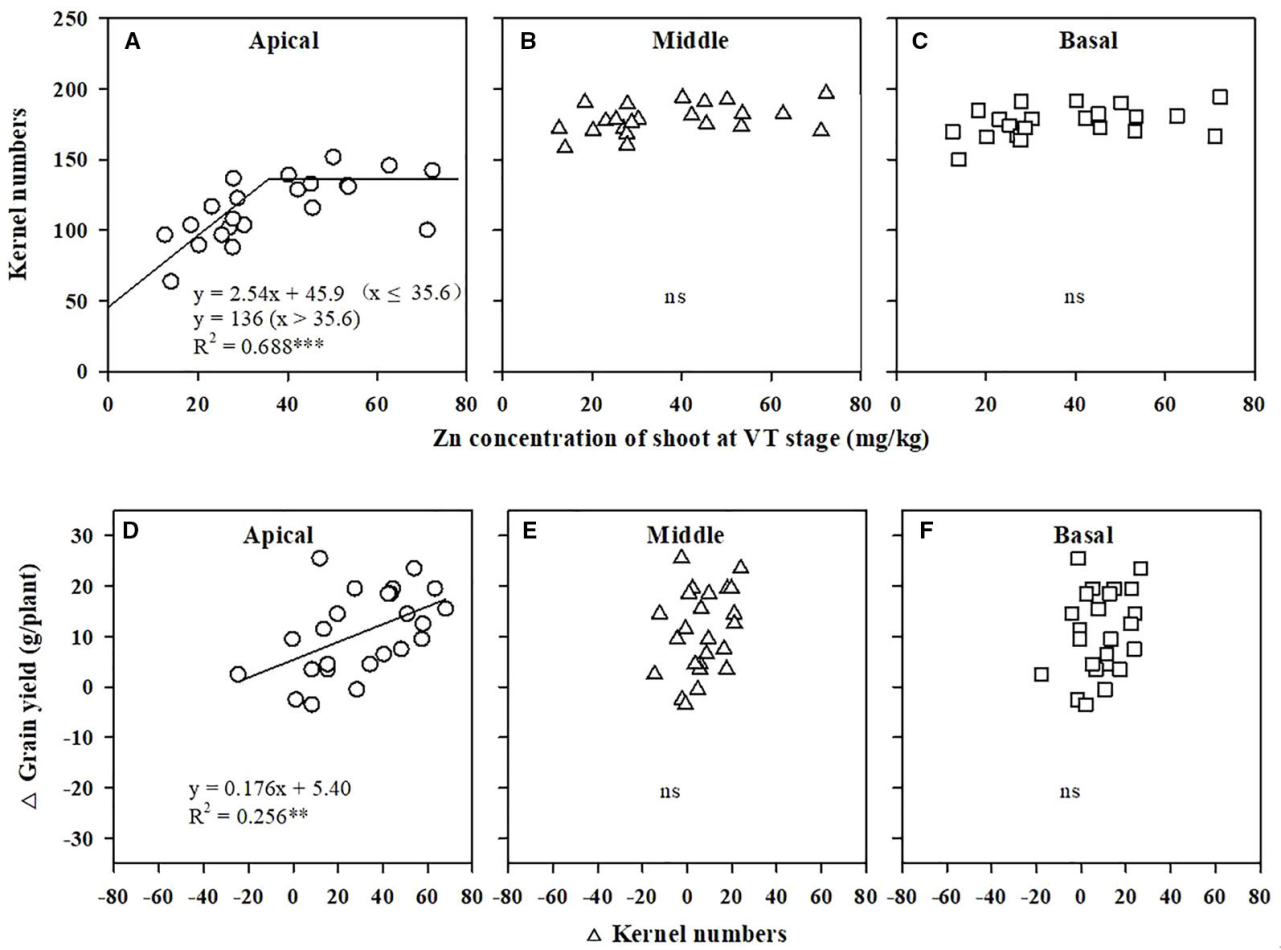
Correlations of kernel numbers in the apical **(A)**, middle **(B)**, and basal **(C)** sections of maize spikes with the Zn concentration in shoots at the tasseling (VT) stage. Correlations of the change in grain yield with the change in kernel numbers of the apical **(D)**, middle **(E)**, and basal **(F)** sections of maize spikes. *** *P* < 0.001, ** *P* < 0.01. ns, not significant.

The KN of the apical section of maize spikes showed a positive linear relation with pollen viability (**Figure 2A**), whereas the KN values of the middle and basal sections were not related to viability (**Figures 2B, C**). Grain yield increased linearly with improved pollen viability (**Figure 2D**). The critical shoot Zn concentration at the VT stage for maximal KN of the apical section was 35.6 mg/kg (**Figure 3A**), whereas the KN values of the middle and basal sections were not related to the shoot Zn concentration (**Figures 3B, C**). The increase in grain yield was positively related to the increase in KN of the apical section of spikes (**Figure 3D**), but was not related to the change in KN of the middle and basal sections (**Figures 3E, F**).

### Effects of Zn Application on Shoot Biomass and Thousand Kernel Weight

**Figure 4:**
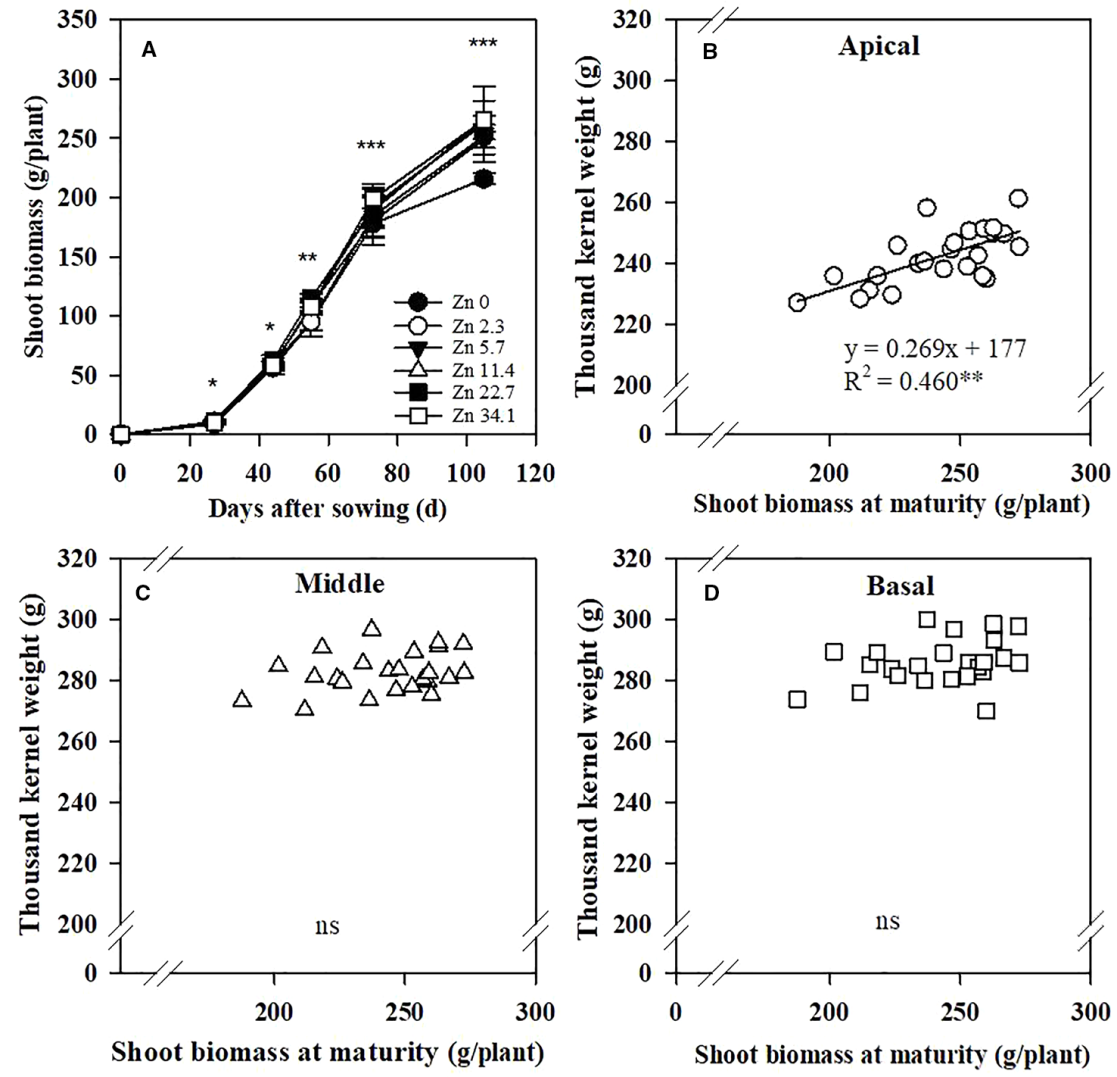
Effects of Zn application rate on shoot biomass at the V6, V12, VT, R3, and R6 stages of maize **(A)** and correlations of the 1,000-kernel weight of apical **(B)**, middle **(C)**, and basal **(D)** sections of maize spikes with shoot biomass at maturity. Shoot biomass refers to the aboveground biomass, which includes stem, leave, and cob after tasseling (VT) stage. Values are the average ± SE of four replicates. ***P < 0.001, **P < 0.01, and *P < 0.05. ns, not significant..

Zinc application significantly increased maize shoot biomass at the V6, V12, VT, R3, and R6 stages, especially in the later growth period (**Figure 4A**). The TKW of the apical section of spikes was positively correlated with the shoot biomass at maturity, but not for the middle and basal sections (**Figures 4B–D**).

### Grain Zn Concentration Is Affected by Zn Application and its Influence on the Kernel Weight of Maize Spike Sections

**Figure 5:**
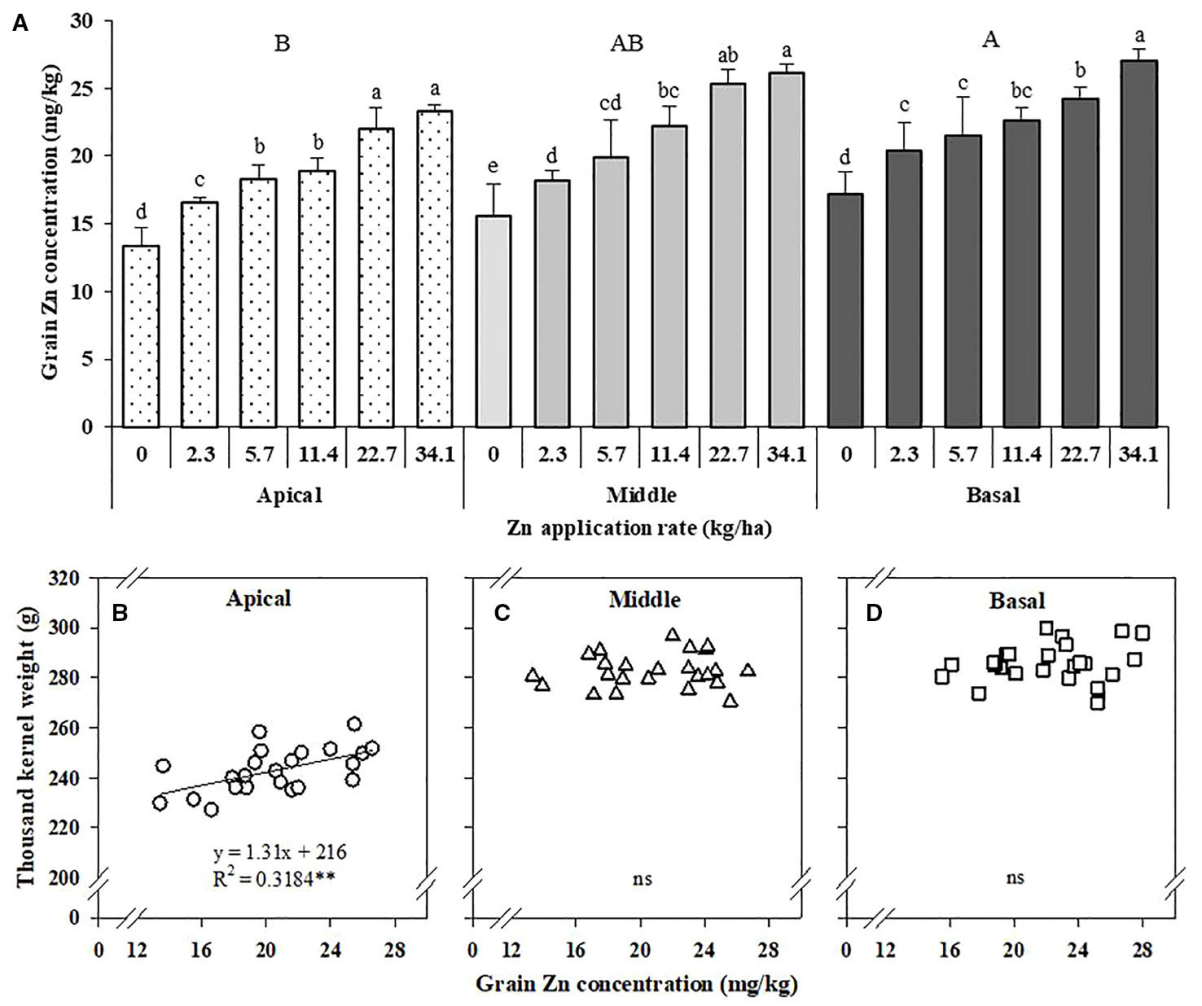
Effects of Zn application on Zn concentration in different sections of maize spikes **(A)** and correlation of the 1,000-kernel weight of the apical **(B)**, middle **(C)**, and basal **(D)** sections of maize spikes with Zn concentration. Values are the average ± SE of four replicates. Within each section means with the same lowercase letters are not significantly different at *P* < 0.05 according to Fisher’s LSD test. The different capital letters indicate a significant difference between sections. ** *P* < 0.01. ns, not significant.

The grain Zn concentration in the apical section of maize spikes was significantly lower than that in the middle and basal sections. With increased Zn application rates, the grain Zn concentrations in all three sections of maize spikes increased significantly (**Figure 5A**). With Zn application, the Zn concentration in the apical section (inferior grains) increased by 24.3–74.9% compared with no Zn treatment (**Figure 5A**). The TKW of the apical section showed a positive linear relationship with Zn concentration in grain, but not for the middle and basal sections (**Figures 5B–D**).

## Discussion

### Improvement of Maize Yield by Zn Application is Mainly Due to Promoting Development of Inferior Grains

Due to the physiological role of Zn in maize, an insufficient supply of Zn reduces maize yield by about 10% (Subedi and Ma, 2009). In our field experiment, soil Zn fertilization increased maize grain yield, which is in agreement consistent with the results reported by Abunyewa and Mercie-Quarshie (2004) and Potarzycki (2010). The explanations provided by these researchers for the increase in maize yield with Zn addition focused mainly on improvements in kernel number and thousand kernel weight. However, those studies measured the kernel number and thousand kernel weight of the entire maize spike (Potarzycki, 2010; Asif et al., 2013) but could not distinguish which section mostly contributed to such improvement. The current study clearly indicated that the improvement of kernel number and thousand kernel weight values in inferior grains explained the increase in maize yield in association with soil Zn application.

### Adequate Zn in Maize Shoots Could Maintain High Pollen Viability and Guarantee Higher Kernel Numbers of Inferior Grains

Pollen viability and the ability of the pistil to produce seeds after pollination are the main factors that determine maize yield (Schoper et al., 1987). Low pollen viability may result in decreasing seed set. Water, heat stress, and nutrient deficiency may also lead to decreased pollen viability (Herrero and Johnson, 1980; Herrero and Johnson, 1981). Zinc application likely promoted pollen viability in the current study, because Zn is essential for pollen grain development, and pollen viability is influenced by many factors, such as relative humidity, temperature, oxygen pressure, etc. (Stanley and Linskens, 2012).

Previous studies have shown that developing anthers and pollen grains have higher Zn requirements than do other plant parts (Sharma et al., 1987), and Zn deficiency may limit these developmental processes. An adequate Zn supply is essential for synthesizing cytoplasmic ribosomes in pollen granulocytes (Prask and Plocke, 1971). Wacker (1962) observed that the RNA and protein levels in Zn-deficient plants were markedly reduced, while excessive polyphosphate and amino acids accumulate.

Furthermore, drought and heat stress in maize are becoming more common during the summer season in the North China Plain (Wang et al., 2018). Zinc plays an important role in alleviating reactive oxygen, drought, and heat stress (Cakmak, 2000; Ma et al., 2017). Thus, adequate Zn in shoots could meet the requirements of pollen development, increase resistance to abiotic stress, and maintain high pollen viability during the anthesis stage.

Currently, the maize planting density widely used in China is below 60,000 plants/ha (Yan et al., 2018). By contrast, the planting density in the US corn belt exceeds 75,000 plants/ha (Yan et al., 2018). Improved maize yield at high planting density is mainly attributable to enhanced kernel numbers and kernel weights. The grain yield of maize is highly correlated with kernel set, which is very sensitive to environmental conditions during the VT stage (Otegui and Bonhomme, 1998). Kernel numbers are related to light interception (Otegui and Andrade, 2000), photosynthesis (Edmeades and Daynard, 1979), and biomass production (Tollenaar and Aguilera, 1992) during critical periods. Adequate Zn in shoots could maintain high pollen viability during the VT stage and increase the grain set.

The current study is the first in which a critical shoot Zn concentration for pollen viability has been established (**Figure 1C**) under high-density field conditions. The suggested critical Zn concentration in shoots for cereal crops is 15–25 mg/kg (Alloway, 2008; Alloway, 2009). However, although values within this range may prevent Zn deficiency in maize, they do not support maximal yield (Sakal et al., 1982; Agrawal, 1992). Previously, we showed that high wheat yields require 29.4 mg/kg Zn (Liu et al., 2017b). In the present study, greater pollen viability required a Zn concentration of 31.2 mg/kg in shoots, and a high kernel number in the apical section of maize spikes required a Zn concentration of 35.6 mg/kg in shoots at the VT stage (**Figures 1C** and **3A**); both of these concentrations are higher than the reported critical Zn deficiency concentrations for cereal crops (Alloway, 2008; Alloway, 2009), and are much closer to our early results for the maximum maize yield requirement, i.e., 33.5 mg Zn kg^−1^ in shoots (Liu et al., 2017a), in a high-density, high-yield system. It should be noted that these values are valid under such meteorological and soil conditions, and that they may change with different climate. We collected meteorological data during the maize growing season from 2007 to 2016 through a meteorological station located next to the experimental filed, and found that the meteorological conditions in 2012 could be representative for last 10 years. Meanwhile, the soil in the current experiment is a typical calcareous alluvial soil in North China Plain. Therefore, the results could be generalized and has practical guidance significance.

### Kernel Weight Improvement of Inferior Grains by Increasing the Shoot Biomass and Grain Zn Concentration

Generally, the formation of maize kernels is limited by the poor distribution of assimilates to the ear (Yoshida, 1972). The growth of kernels on the apical section is initiated 4–5 days after that of the basal kernels (Tollenaar and Daynard, 1978). Maize inferior grain usually has low thousand kernel weight values because of the shorter period of grain filling and the lower grain filling rate of kernels in the apical section (Tollenaar and Daynard, 1978; Hanft et al., 1986). Improved sink activity, sources, and assimilate and nutrient flow are essential to increasing yield. The poor filling of inferior grains might be caused by an inadequate carbohydrate supply (Hanft et al., 1986). In the current study, the biomass of maize across the growth period increased with Zn application. This means that Zn application provided adequate carbohydrates, representing a “source”. Thus, Zn application enhanced the thousand kernel weight of maize inferior grain and guaranteed the grain-filling process.

Increasing the Zn concentration in maize grain through biofortification is important for human and livestock feeding (Cakmak, 2008). In the present study, the Zn concentration of inferior grain increased from 13.3 to 23.3 mg/kg with 34.1 kg/ha Zn application, while the target value suggested by the USDA Nutrient Database (http://ndb.nal.usda.gov/ndb/) is 22.1 mg/kg. Molecular mapping of the grain Zn concentration and thousand kernel weight in wheat showed a strong positive association between Zn and thousand kernel weight, suggesting that improving one of the traits allows the other to improve simultaneously (Krishnappa et al., 2017). The increasing grain Zn concentration improved the thousand kernel weight of maize spikes (Ziaeyan and Rajaie, 2012), and the current study indicates that this happens in the inferior grains.

The average grain Zn concentrations in the middle and basal parts of maize spike were higher than that in the apical part. Unlike this result, some studies observed no significant differences in mineral elements between inferior and superior grains [e.g., (Mozafar, 1990)]. One possible explanation for this discrepancy is that the current study examined several Zn application rates, whereas Mozafar (1990) only added N, P, and K. Because of the limited retranslocation of resources, the Zn concentration might not be improved as much in inferior as in superior grains after Zn application.

## Conclusion

In Zn-deficient soils, Zn application increased maize yield due to increased kernel numbers and kernel weight in inferior grains. An adequate Zn supply in maize plants maintained high pollen viability and a sufficient carbohydrate source. The critical shoot Zn concentrations for high pollen viability and high kernel numbers of inferior grains were 31.2 and 35.6 mg/kg, respectively.

## Data Availability Statement

The datasets generated for this study are available on request to the corresponding author.

## Author Contributions

X-PC and C-QZ conceived and designed the experiments. D-YL, Y-ML, and WZ performed the experiments. D-YL and X-PC analyzed the data and wrote the paper.

## Funding

This research was funded by grants from the National Key Research and Development Program of China (2018YFD0200701), the National Maize Production System in China (CARS-02-24), the National Science Foundation of China (31672240), and the State Cultivation Base of Eco-agriculture for Southwest Mountainous Land (Southwest University).

## Conflict of Interest

The authors declare that the research was conducted in the absence of any commercial or financial relationships that could be construed as a potential conflict of interest.
